# Non-interventional follow-up versus fluid bolus in RESPONSE to oliguria in hemodynamically stable critically ill patients: a randomized controlled pilot trial

**DOI:** 10.1186/s13054-022-04283-8

**Published:** 2022-12-22

**Authors:** Nina Inkinen, Ville Pettilä, Miia Valkonen, Maija Serlo, Minna Bäcklund, Johanna Hästbacka, Anni Pulkkinen, Tuomas Selander, Suvi T. Vaara

**Affiliations:** 1 Department of Anesthesia and Intensive Care, Central Finland Hospital Nova, Central Finland Health Care District, Hoitajantie 3, 40620 Jyväskylä, Finland; 2 Division of Intensive Care Medicine, Department of Perioperative, Intensive Care and Pain Medicine, University of Helsinki and Helsinki University Hospital, Helsinki, Finland; 3 Science Service Center, Kuopio University Hospital, Kuopio, Finland

**Keywords:** Oliguria, Fluid bolus, Fluid balance, Sepsis, Acute kidney injury

## Abstract

**Background:**

Fluid bolus therapy is a common intervention to improve urine output. Data concerning the effect of a fluid bolus on oliguria originate mainly from observational studies and remain controversial regarding the actual benefit of such therapy. We compared the effect of a follow-up approach without fluid bolus to a 500 mL fluid bolus on urine output in hemodynamically stable critically ill patients with oliguria at least for 2 h (urine output < 0.5 mL/kg/h) in randomized setting.

**Methods:**

We randomized 130 patients in 1:1 fashion to receive either (1) non-interventional follow-up (FU) for 2 h or (2) 500 mL crystalloid fluid bolus (FB) administered over 30 min. The primary outcome was the proportion of patients who doubled their urine output, defined as 2-h urine output post-randomization divided by urine output 2 h pre-randomization. The outcomes were adjusted for the stratification variables (presence of sepsis or AKI) using two-tailed regression. Obtained odds ratios were converted to risk ratios (RR) with 95% confidence intervals (CI). The between-group difference in the continuous variables was compared using mean or median regression and expressed with 95% CIs.

**Results:**

Altogether 10 (15.9%) of 63 patients in the FU group and 22 (32.8%) of 67 patients in FB group doubled their urine output during the 2-h period, RR (95% CI) 0.49 (0.23–0.71), *P* = 0.026. Median [IQR] change in individual urine output 2 h post-randomization compared to 2 h pre-randomization was − 7 [− 19 to 17] mL in the FU group and 19[0–53] mL in the FB group, median difference (95% CI) − 23 (− 36 to − 10) mL, *P* = 0.001. Median [IQR] duration of oliguria in the FU group was 4 [2–8] h and in the FB group 2 [0–6] h, median difference (95%CI) 2 (0–4) h, *P* = 0.038. Median [IQR] cumulative fluid balance on study day was lower in the FU group compared to FB group, 678 [518–1029] mL versus 1071 [822–1505] mL, respectively, median difference (95%CI) − 387 (− 635 to − 213) mL, *P* < 0.001.

**Conclusions:**

Follow-up approach to oliguria compared to administering a fluid bolus of 500 mL crystalloid in oliguric patients improved urine output less frequently but lead to lower cumulative fluid balance.

*Trial registration* clinical.trials.gov, NCT02860572. Registered 9 August 2016.

**Graphical Abstract:**

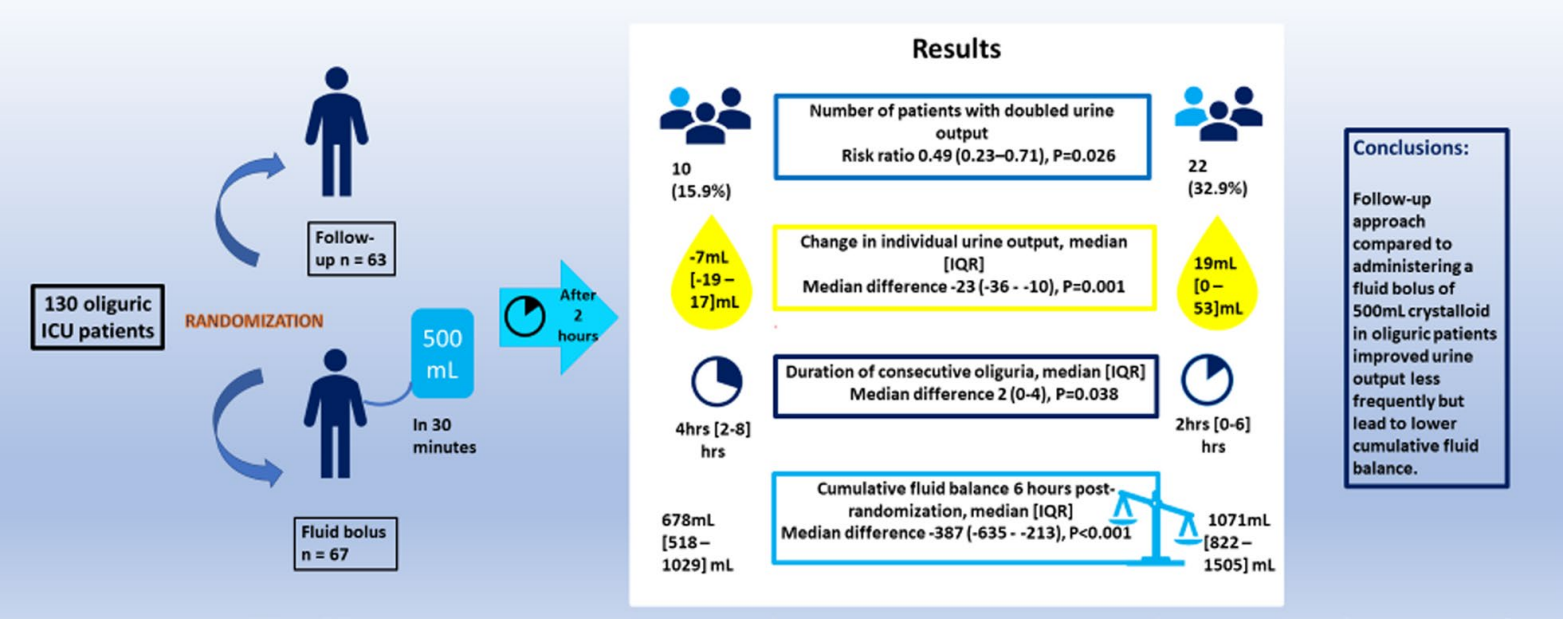

**Supplementary Information:**

The online version contains supplementary material available at 10.1186/s13054-022-04283-8.

## Background

Oliguria is a common disorder in critically ill patients [1, 2], and fluid bolus therapy is a frequent intervention aimed at increasing urine output [3–7]. Few studies have examined the effect of a fluid bolus on urine output [8–11] and found that relatively few patients actually increase their urine output after receiving a fluid bolus. Fluid boluses are administered to improve cardiac output and, subsequently, ensure sufficient renal blood flow and tissue perfusion. A typically administered fluid bolus has been 500 mL of crystalloid [3, 12]. However, several studies have shown poor correlation between systemic hemodynamics and renal response [8, 9, 13]. Moreover, evidence is accumulating about the harms of fluid accumulation such as increased risk for the development of acute kidney injury (AKI) [14–17] and mortality [14, 15, 17–22]. Additionally, excessive fluid may decelerate renal recovery [23] or worsen AKI [17, 24–26].

One possible approach to reduce the accumulation of fluid would be avoiding administering extra fluid. Earlier results concerning the effect of a fluid bolus on urine output are based on observational studies [8, 10, 11] and post hoc analyses of randomized trials [9]. Therefore, we conducted a randomized controlled pilot trial to examine the effect of a follow-up (FU) compared to fluid bolus (FB) on urine output in critically ill oliguric patients.

## Methods

### Trial design

We conducted an investigator-initiated, open, randomized, controlled pilot trial (the RESPONSE trial) (clinicaltrials.gov registry identifier NCT02860572) in two intensive care units (ICUs) at Meilahti Hospital (Helsinki University Hospital) and at Central Finland Central Hospital (Jyväskylä). The trial protocol and statistical analysis plan have been published [27]. The Ethics Committee of the Department of Surgery, Hospital District of Helsinki and Uusimaa, approved the trial (Decision number HUS/1308/2016). The trial was conducted according to Declaration of Helsinki and its later amendments and according to Good Clinical Practice guidelines. Because of the critically ill patient population and a time-sensitive intervention, a deferred consent was approved with an informed, written consent obtained from the patient or patient’s next of kin as soon as possible.

We randomized patients to either FU or FB group with an allocation ratio of 1:1. Randomization was stratified according to the presence/absence of sepsis using the Sepsis-3 definition [28] and AKI defined by KDIGO criteria [29]. An independent statistician created a computer-based algorithm, and we used a web-based allocation concealment (Absolute Imaginary Software Ltd., Kauniainen, Finland) for randomization and data collection. Permuted blocks of varying size (4, 6 or 8) were used. The allocation was blinded for the person conducting the data analysis. Because of the nature of the trial, blinding of the ICU personnel was not feasible.

### Patients

**Fig. 1:**
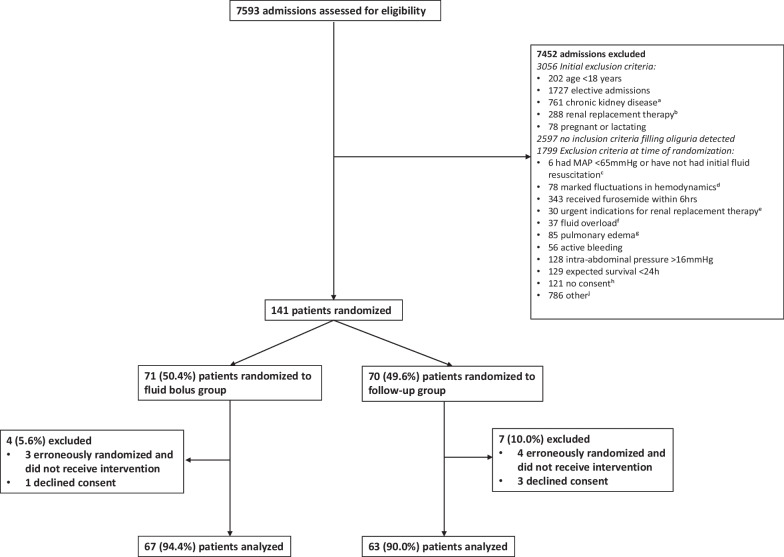
Flowchart. ^a^Chronic kidney disease (estimated precritical illness glomerular filtration rate < 60 mL/min/1.73 m^2^). ^b^Renal replacement therapy (RRT) has been already started in the ICU for AKI, or commencing RRT (according to last laboratory values) was likely within the next 6 h or patient underwent regular (chronic) dialyses. ^c^Patient has received less than 20 mL/kg i.v. fluids during the last 12 h for shock/hypovolemia or patient has been in the ICU less than 6 h. ^d^Cardiac arrhythmias affecting blood pressure, increase in norepinephrine need over 0.2 ug/kg/min, need for initiation of inotrope/inodilator within the last 2 h pre-randomization. ^e^Urgent indications for commencing RRT for AKI at the moment of randomization were present (based on last blood work): plasma potassium > 6 mmol/L or severe metabolic acidosis (pH < 7.20 and bicarbonate < 12 mmol/L) or evidence of severe respiratory failure (PaO_2_/FiO_2_ ratio < 200) and clinical perception of volume overload or AKI has continued over 72 h. (Creatinine remains more than twice the normal level/oliguria continued.) ^f^Cumulative fluid accumulation exceeds 10% of baseline body weight. ^g^Bilateral infiltrates in chest X-ray. ^h^Obtaining informed written consent was not possible (i.e., patient or her/his next of kin did not speak Finnish or Swedish), or consent was denied. ^j^For example organ recipients, cardiopulmonary resuscitated patients with temperature control treatment, severe electrolyte disturbances (predominantly hyponatremia), logistic reasons, patients recruited in another study. Patient recruitment was interrupted in 2020 from March to May because of COVID-19 pandemic

We screened all patients at admission to participating ICUs during study period for initial eligibility using the following criteria: (1) 18 year or older, (2) emergency admission, (3) no chronic kidney disease (estimated precritical illness GFR > 60 mL/ min/1.73 m2), (4) no chronic renal replacement therapy (RRT) or urgent need for RRT, and (5) not pregnant or lactating. Patients were eligible until 72 h from ICU admission. Of those who were initially eligible, we enrolled patients who developed oliguria (urine output < 0.5 mL/kg/h) lasting at least two consecutive hours (e.g., from 1 to 2 pm continuing from 2 to 3 pm) and did not fulfill any of the exclusion criteria at the time of randomization (Fig. 1). We included only hemodynamically stable patients to minimize the risk for the need of extra fluid boluses in the FU group. Thus, patients, e.g., with marked fluctuations in hemodynamics or active bleeding were excluded (Additional file 1: Table S1). All patients had urine catheter in place and urine output was recorded hourly. Patients were considered hemodynamically stable if they (1) had mean arterial pressure (MAP) over 65 mmHg with or without vasopressors, and (2) had been in the ICU at least 6 h and thus presumably received appropriate initial fluid resuscitation, or they had received at least 20 mL/kg fluids and were not actively bleeding. Details of inclusion and exclusion criteria are presented in Additional file 1: Table S1. Randomization occurred mainly between 7 am and 10 pm due to the availability of study personnel.

### Intervention

Patients in the FU group did not receive fluid bolus or diuretics to increase urine output during the 2-h study period. Patients in the FB group received 500 mL balanced crystalloid (Ringer’s acetate) infused over 30 min. In both groups, all ongoing infusions such as nutrition and maintenance fluid were infused constant during the 2-h period. Vasoactive drugs, insulin, sedation, and other medications were titrated according to the judgment of the treating clinician, but MAP target level was not modified. Diuretics were not allowed during the 2-h study period. In case of severe hemodynamic instability (need to increase norepinephrine-infusion > 0.2 μg/kg/min from baseline) or heart rate increase > 30 beats/min from baseline (due to suspected hypovolemia), a rescue bolus of 500 mL crystalloid over 30 min was allowed in both groups according to the decision of the treating clinician. Urine output was recorded hourly until 6 h post-randomization.

### Outcomes

The primary outcome was the number of patients who doubled their urine output using the following definition: the mean cumulative 2-h urine output (mL/kg/h) 2 h randomization divided by the mean cumulative 2-h urine output (mL/kg/h) measured 2 h preceding randomization expressed as percentage. Based on a previous prospective cohort study, we considered doubling of the urine output as a clinically significant increase in urine output among oliguric patients [30], if urine output increased at least 10 mL/h (i.e., difference between cumulative 2-h urine output post-randomization versus pre-randomization was at least 20 mL).

The secondary outcomes included the change in individual urine output, duration of consecutive oliguria (urine output < 0.5 mL/kg, hours from randomization), and cumulative fluid balance on study day (6 h from randomization).

The exploratory outcomes included physiological effects [i.e., MAP, heart rate, norepinephrine dose, central venous pressure (CVP), difference in core vs. peripheral temperature, capillary refill time (until 60 min), arterial blood pH, standard base excess, arterial lactate] during 2-h period from randomization, number of patients receiving rescue boluses and the number of rescue boluses, number of patients with protocol violations, number of patients with adverse events, highest AKI stage within 24 h, 48 h, and during ICU stay and number of patients receiving RRT. Definitions for protocol violations and adverse events are listed in Additional file 1: Table S2.

### Statistical analysis

The detailed statistical analysis plan has been published [27]. Previous data to inform about the incidence of the primary outcome were inconsistent [8, 9], but we assumed that 30% in the FB group would have a positive primary endpoint [27]. To reach a 20% absolute difference in the primary outcome that we consider as the minimum clinically meaningful difference for this frequent clinical intervention, 62 patients per group would be required to reach 80% power with two-sided significance level set at 0.05. Eventually, we chose to randomize 65 patients per group considering replacement of possible dropouts.

We performed the primary analyses on the modified intention-to-treat (ITT) population defined as all randomized patients excluding patients without consent and patients who were erroneously randomized and did not receive the trial intervention. A sensitivity analysis was performed on the per-protocol population, i.e., ITT population excluding patients who experienced protocol violation(s) or received a rescue bolus.

The outcome variables were adjusted for the stratification variables as recommended [31], i.e., sepsis according to sepsis-3 definition [28] or AKI defined by the KDIGO criteria [29] using two-tailed logistic regression. Obtained odds ratios (OR) were converted to risk ratios (RR) with 95% confidence intervals (CI). Additionally, we conducted a crude analysis for the primary and secondary outcomes. Regarding the exploratory physiological outcomes, only crude analysis was conducted. The between-group difference in the continuous variables was compared using mean or median regression depending on normal distribution and expressed with 95% CIs. Dichotomous variables were analyzed using logistic regression and reported as RRs with 95% CIs. Group differences on repeated measurements were compared by linear mixed effect model. Normally distributed variables were analyzed on original scale, and results were expressed as means with standard errors of means (SEM). Skewed variables were log-transformed before linear mixed effect model analyses, and for these variables, results were expressed as geometric means with 95% CIs. We tested continuous variables for normality using the Shapiro–Wilk test. We performed the analysis using R statistical software version 3.6.2 and SPSS statistics 27. External staff monitored trial data including informed consent, inclusion and exclusion criteria, and from a randomly selected subset of patients all source data.

## Results

### Patients

Between January 2017 and November 2020, we screened 7593 admissions for initial eligibility. Altogether 4537 (59.8%) fulfilled the initial inclusion criteria, and finally, 1940 (25.5%) patients were observed to develop oliguria. After exclusions (Fig. 1), 141 (7.3%) patients were randomized. Furthermore, we excluded four (2.8%) patients who declined consent and seven (5.0%) patients who did not fulfill the oliguria inclusion criterion but were erroneously randomized and did not receive the trial intervention. Thus, we included 130 patients in the modified ITT analysis. Altogether 63 (48.5%) patients were randomized to FU group and 67 (51.5%) to FB group. Patient baseline characteristics were well balanced (Table 1). The most frequent ICD-10 ICU admission diagnoses were sepsis (10, 7.7%), ruptured abdominal aortic aneurysm (10, 7.7%), acute vascular disorders of the intestine (6, 4.6%), and pneumonia (6, 4.6%).

**Table 1:** Patient characteristic in intervention groups

	Follow-up group, *n* = 63	Fluid bolus group, *n* = 67
Age (years)	67 [53–72]	69 [59–76]
Sex; female (%)	19 (30.2)	26 (38.8)
Weight (kg)	85 [75–102]	83 [73–100]
Hypertension (%)	37 (58.7)	44 (65.7)
Chronic heart failure (%)	1 (1.6)	2 (3.0)
Atrial fibrillation (%)	9 (14.3)	14 (20.9)
Coronary artery disease (%)	4 (6.3)	7 (10.4)
Arteriosclerosis obliterans (%)	4 (6.3)	5 (7.5)
Chronic obstructive pulmonary disease (%)	5 (7.9)	4 (6.0)
Chronic liver insufficiency (%)	2 (3.2)	0 (0.0)
Diabetes (%)	13 (20.6)	22 (32.8)
Malignancy (%)	12 (19.0)	9 (13.4)
Rheumatoid disease (%)	4 (6.3)	4 (6.0)
SAPS II score	37 [34–45]	41 [33–48]
Surgical admission (%)	33 (52.4)	37 (55.2)
*At randomization*		
Time from ICU admission to randomization (hours)	18.8 [11.1–29.2]	20.2 [12.5–35.2]
Sepsis (%)	35 (55.6)	37 (55.2)
Acute kidney injury (%)	37 (58.7)	40 (59.7)
Acute kidney injury stage 1 (%)*	19 (51.4)	25 (62.5)
Acute kidney injury stage 2 (%)*	11 (29.7)	11 (27.5)
Acute kidney injury stage 3 (%)*	7 (18.9)	4 (10.0)
Invasive ventilation (%)	33 (52.4)	34 (50.7)
Vasoactive medication (%)**	30 (47.6)	32 (47.8)
Continuous sedation (%)	33 (52.4)	28 (41.8)
Cumulative balance from ICU admission to randomization (mL)	1856 [1296–2948]	1941 [1201–3762]
SOFA score at randomization (− 24 to 0 h)	7 [6–10]	7 [5–9]
SOFA score 24 h post-randomization (0 to 24 h)	7 [5–8]***	6 [3–9]

Data included from all 130 patients

Categorical data reported as count (percentage) and continuous data as median [interquartile range, IQR]

*ICU* intensive care unit, *SAPS* Simplified Acute Physiology Score II, *SOFA* Sequential Organ Failure Assessment; considering all six organ systems

*According to KDIGO criteria

**Norepinephrine, dobutamine, epinephrine, levosimendan, milrinone, vasopressin, dopamine, or other

***Data missing from one patient

### Primary and secondary outcomes

**Fig. 2:**
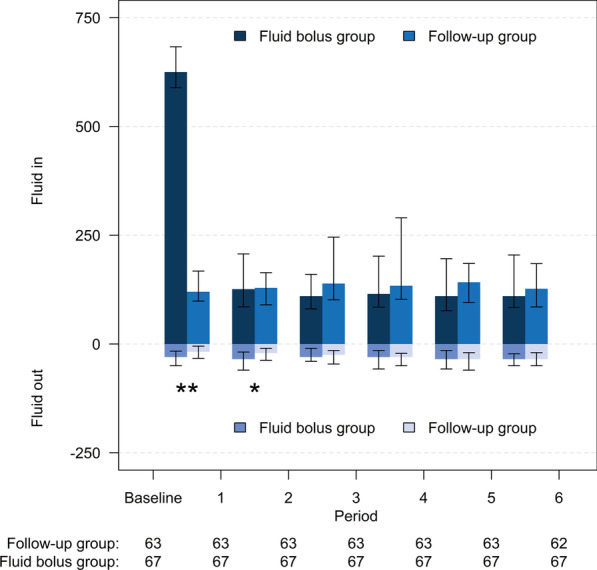
Administered fluids and urine output 6 h post-randomization according to intervention group. Bars represent median and whiskers interquartile range. *P < 0.05 and **P < 0.01, adjusted with sepsis and AKI; urine output did not differ from 3 h onward. Fluids in (mL) consisted of Ringer bolus (only in FB group), maintenance fluids, nutrition, medication, blood products, and possible rescue bolus. Fluid out (mL) consisted of urine output only. Period is expressed as hours

Table 2 reports the primary and secondary outcomes. Altogether 10 (15.9%) patients in the FU group and 22 (32.9%) patients in the FB group doubled their urine output (difference between the groups 17 absolute percentage points), risk ratio (95% CI) 0.49 (0.23–0.71), *P* = 0.026. The duration of improved urine output (> 0.5 mL/kg/h) was 2 h in the FB group (Fig. 2). Median [IQR] change in urine output 2 h post-randomization compared to 2 h pre-randomization was -7 [-19–17] mL in the FU group and 19 [0–53] mL in the FB group, median difference (95% CI) − 23 (− 36 to − 10) mL, *P* = 0.001. Duration of consecutive oliguria after randomization was longer in the FU group than in the FB group, median [IQR] 4 [2–8] h versus 2 [0–6] h, median difference (95% CI) 2 (0–4) h, *P* = 0.038. The cumulative fluid balance on the study day was lower in the FU group compared to FB group, median [IQR] 678 [518–1029] mL versus 1071 [822–1505] mL, median difference (95%CI) − 387 (− 635 to − 213) mL, *P* < 0.001. In the crude analysis without adjustment for stratification variables, results were unchanged except for duration of oliguria being not different (Additional file 1: Table S3). In a *post hoc* analysis among FB group patients, we found no differences in patient characteristics between patients who doubled their urine output and patients who did not (Additional file 1: Table S4). Median [IQR] change in urine output 2 h post-randomization compared to 2 h pre-randomization was 76 [51–108] mL in patients who doubled urine output and 10 [− 10 to 19] mL in patients who did not, median difference (95% CI) 73 (39–90) mL, *P* < 0.001.

**Table 2:** Primary and secondary outcomes

	Follow-up group, *n* = 63	Fluid bolus group, *n* = 67	Follow-up group vs Fluid bolus group (95% CI)	*P *value^c^
*Primary outcome*				
Number of patients with doubled urine output (%)	10 (15.9)	22 (32.9)	0.49* (0.23–0.71)	0.026
*Secondary outcomes*				
Change in individual urine output, median [IQR], mL	− 7 [− 19 to 17]	19 [0–53]	− 23** (− 36 to − 10)	0.001
Duration of consecutive oliguria, median [IQR], hours^a^	4 [2–8]	2 [0–6]	2** (0–4)	0.038
Cumulative fluid balance 6 h post-randomization, median [IQR], mL^b^	678 [518–1029]	1071 [822–1505]	-387** (− 635 to − 213)	< 0.001

Data included from all 130 patients

*Risk ratio

**Median difference

^a^Urine output < 0.5 mL/kg/h, data collected to 30d post-randomization or ICU discharge if earlier

^b^Including fluid input and urine output

^c^Adjusted (sepsis and acute kidney injury) median difference or risk ratio with 95% CIs

### Exploratory outcomes

**Fig. 3:**
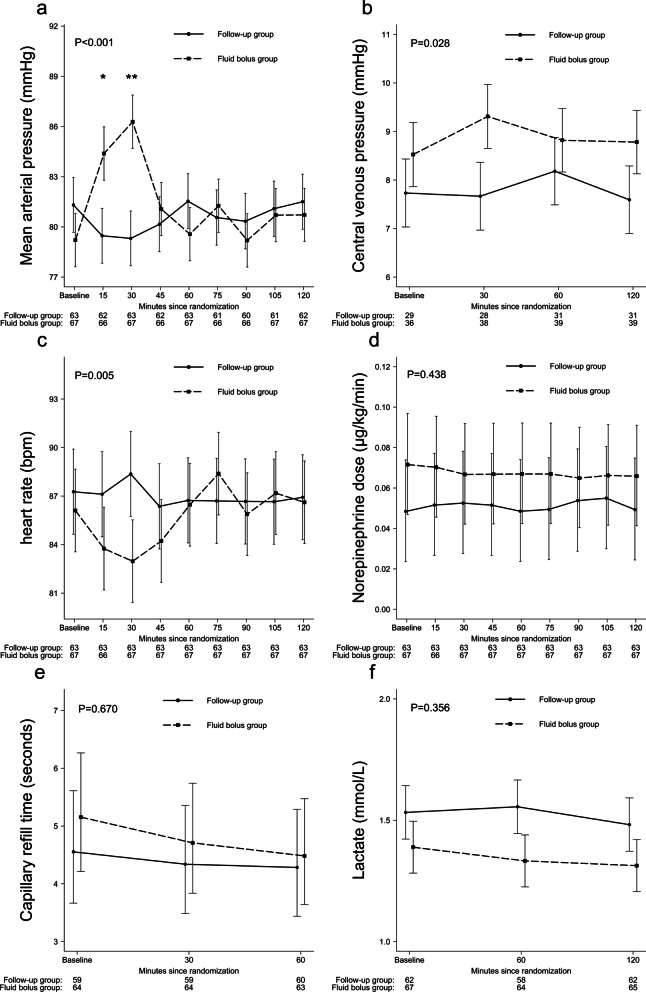
**a** Mean arterial pressure, **b** central venous pressure, **c** heart rate, **d** norepinephrine dose, **e** capillary refill time and **f** arterial lactate from 0 to 6 h. The difference between FU and FB group was compared with linear mixed effect model. Values are means with SEM in figures (**a**, **b**, **c** and **f)**. In figures **d** and **e**, values are geometric means with 95% Cis. *P < 0.05 and **P < 0.01 between groups on time points

We found MAP to be lower and heart rate higher in FU group compared to FB group at the first 15 and 30 min from randomization (Fig. 3). This difference dissipated after the first hour (Fig. 3). CVP was lower in the FU group compared to FB group (Fig. 3). The groups did not differ in terms of norepinephrine dose, difference in core vs peripheral temperature, capillary refill time, arterial pH, arterial base excess, or lactate during the 2-h follow-up (Fig. 3, Additional file 1: Fig. S1). We found no differences in the highest AKI stage between the groups (Additional file 1: Table S5). All results of the non-physiological exploratory outcomes are presented in Additional file 1: Table S5.

### Subgroup analyses

In the subgroup of septic patients (*n* = 72; 35 in the FU and 37 in the FB group), we found 5 (14.3%) patients in the FU group and 10 (27.0%) patients in the FB group to double their urine output, risk ratio (95% CI) 0.54 (0.19–1.34), *P* = 0.198, (Additional file 1: Table S6). In the subgroup of patients with AKI at randomization (*n* = 77; 37 in the FU group 40 in the FB group), the primary endpoint occurred in 5 (13.5%) patients in the FU group and in 10 (25.0%) patients in the FB group, risk ratio (95%CI) 0.56 (0.19–1.40), *P* = 0.222, (Additional file 1: Table S7). Cumulative fluid balance on the study day was lower in the FU group compared to the FB group in both subgroups (Additional file 1: Tables S6, S7).

### Per-protocol patient population analysis

In the per-protocol patient population analyses, the results were comparable as in the modified ITT population analysis, except regarding the duration of consecutive oliguria results (Additional file 1: Table S8).

## Discussion

We conducted a randomized controlled trial among 130 critically ill oliguric patients to compare a 2-h non-interventional follow-up approach to a fluid bolus of 500 mL crystalloid infused over 30 min to increase urine output. Fewer patients in the FU group doubled their urine output in the 2-h follow-up compared to FB group; however, this outcome occurred in only one-third of patients in the FB group. Individual urine output increased less in FU group compared to the FB group, but the effect in the FB group was small and short-lived. Additionally, the duration of oliguria was longer in the FU group compared to FB group, but the difference between the groups was only 2 h. Importantly, the cumulative fluid balance on the study day was lower in the FU group compared to the FB group.

We compared the effect of a FU approach to a typical clinical response to oliguria in the critically ill, namely a fluid bolus of 500 mL administered over 30 min [3]. In a multicenter observational trial, oliguria was the second most common reason to administer such a bolus [3], although the supporting evidence is weak [12] and the response to this intervention remains poorly registered in clinical practice [3]. Among mixed critically ill patients, observational studies [8, 9] have not detected an association with fluid bolus and improved urine output, whereas an interventional study among patients with circulatory shock found urine output to roughly double after a large fluid bolus [10]. Our trial patients were hemodynamically stable and had received the initial fluid resuscitation which may explain the more distinct effect of a fluid bolus on oliguria in the study by Moussa et al. [10]. Moreover, according to previous studies the effect of fluid bolus on hemodynamics is short-lived [32, 33] which our trial confirmed in a randomized setting.

Urine output less than 0.5 mL/kg/h for 6 h is considered as one of the criteria of AKI and oliguria [29] even though more strict thresholds have been proposed [34]. We chose to use doubling of urine output as the primary outcome, as this outcome had been previously used in a pharmacological study [30]. However, we acknowledge that this outcome is arbitrary, and it can become “positive” even though the actual increase in urine output would be very small. Therefore, we reported also the change in urine output and the duration of oliguria. All these outcomes signaled that patients in the FU group produced slightly smaller amounts of urine; however, the urine output response in the FB group was very modest considering that the fluid bolus volume was 500 mL. Consequently, the fluid balance in the FB group significantly increased. Furthermore, the urine output between the groups was similar from 3 h after the intervention onward indicating a very short improvement in the urine output. The results in the subgroups of septic patients and AKI patients corroborated those of the main analysis. As this was a pilot trial concentrating on physiological effects of fluid bolus, the trial was not powered to detect differences in patient-centered outcomes such as duration of AKI or survival. Our trial did not reveal harms related to smaller amount of given fluid. Follow-up approach on oliguria was feasible and well tolerated. Notably, another pilot trial among patients with AKI found a restricted approach to fluid therapy aiming at neutral fluid balance to be safe [35]. Considering the harms of fluid accumulation, it is unlikely that critically ill, hemodynamically stable patients with oliguria benefit from fluid bolus therapy administered to correct oliguria. Importantly, the FU approach did not associate with worsening of physiological parameters, acid–base balance, or more severe AKI. Finally, AKI is a heterogeneous syndrome [36] and therefore tailoring fluid therapy according the individual course of disease, coexisting comorbidities, other ICU syndromes and ongoing therapies might be the best approach.

### Study strengths and limitations

Our trial has several strengths. First, randomized controlled setting to minimize selection bias is an obvious strength in assessing this study question, while previous data come mostly from observational and registry-based studies. Second, we enrolled patients from both tertiary and central hospitals. Third, our primary outcome was objectively measurable and therefore not exposed to bias. Fourth, external staff monitored the trial data (Additional file 2).

This trial has also some limitations. First, we screened a large number of patients and every fourth of them developed oliguria, and it is likely that some eligible patients were left unrecognized. However, this occurred randomly and improbably caused any selection bias. Second, ICU personnel were not blinded to the intervention. Third, the trial patients were monitored according to normal ICU practices as indicated by their clinical status and according to treating clinician’s consideration. We did not have any standardized protocol for assessing fluid responsiveness or volume status and most patients did not have cardiac output monitoring. Therefore, we cannot comment whether the trial patients were fluid responsive. However, only patients who had received the initial fluid resuscitation and maintained adequate mean arterial pressure (with or without vasopressor) were enrolled. Additionally, the correlation between hemodynamics and urine output is inconsistent [8, 10]. Fourth, decent proportion of ICU admissions was excluded (e.g., elective admissions, patients with chronic kidney disease) and our results are not generalizable in these patient groups. Fifth, we mostly recruited patients between 7 am. and 10 pm., which limits generalizability. However, under normal physiological conditions, urine production is reduced during the night time, and the effect of a fluid bolus on urine output may have been even smaller in the night time [37].

## Conclusions

Follow-up approach to oliguria compared to a fluid bolus of 500 mL crystalloid improved urine output less frequently but leads to a lower cumulative fluid balance. Overall, the duration of oliguria was short. The benefits of fluid bolus therapy over the potential harms associated with fluid accumulation in oliguric critically ill patients should be carefully considered.

## Supplementary Information

**Additional file 1.** Supplemental Tables and Figures.

**Additional file 2.** Dataset.

## Data Availability

The dataset supporting the conclusions of this article is included within the article and its additional files.

## Abbreviations

AKI: Acute kidney injury
CI: Confidence interval
CVP: Central venous pressure
FB: Fluid bolus
FU: Follow-up
GFR: Glomerular filtration rate
ICU: Intensive care unit
IQR: Interquartile range
ITT: Intention-to-treat
MAP: Mean arterial pressure
OR: Odds ratio
RR: Risk ratio
RRT: Renal replacement therapy
SAPS II: Simplified Acute Physiology Score II
SOFA: Sequential Organ Failure assessment
SEM: Standard errors of means

## References

[1] Vincent JL, Ferguson A, Pickkers P, Jakob SM, Jaschinski U, Almekhlafi GA (2020). The clinical relevance of oliguria in the critically ill patient: analysis of a large observational database. Crit Care Lond Engl.

[2] Vaara ST, Parviainen I, Pettilä V, Nisula S, Inkinen O, Uusaro A (2015). Association of oliguria with the development of acute kidney injury in the critically ill. Kidney Int.

[3] Cecconi M, Hofer C, Teboul JL, Pettila V, Wilkman E, Molnar Z (2015). Fluid challenges in intensive care: the FENICE study: a global inception cohort study. Intensive Care Med.

[4] Messina A, Longhini F, Coppo C, Pagni A, Lungu R, Ronco C (2017). Use of the fluid challenge in critically Ill adult patients: a systematic review. Anesth Analg.

[5] Glassford NJ, Mårtensson J, Eastwood GM, Jones SL, Tanaka A, Wilkman E (2016). Defining the characteristics and expectations of fluid bolus therapy: a worldwide perspective. J Crit Care.

[6] Bihari S, Prakash S, Bersten A (2013). Post resusicitation fluid boluses in severe sepsis or septic shock: prevalence and efficacy (price study). Shock [Internet].

[7] Bjerregaard MR, Hjortrup PB, Perner A (2019). Indications for fluid resuscitation in patients with septic shock: post-hoc analyses of the CLASSIC trial. Acta Anaesthesiol Scand.

[8] Legrand M, Le Cam B, Perbet S, Roger C, Darmon M, Guerci P (2016). Urine sodium concentration to predict fluid responsiveness in oliguric ICU patients: a prospective multicenter observational study. Crit Care Lond Engl.

[9] Lammi MR, Aiello B, Burg GT, Rehman T, Douglas IS, Wheeler AP (2015). Response to fluid boluses in the fluid and catheter treatment trial. Chest.

[10] Moussa MD, Scolletta S, Fagnoul D, Pasquier P, Brasseur A, Taccone FS (2015). Effects of fluid administration on renal perfusion in critically ill patients. Crit Care Lond Engl.

[11] Felice VB, Lisboa TC, de Souza LV, Sell LC, Friedman G (2010). Pacientes oligúricos hemodinamicamente estáveis geralmente não respondem ao desafio hídrico. Rev Bras Ter Intensiva [Internet].

[12] Glassford NJ, Eastwood GM, Bellomo R (2014). Physiological changes after fluid bolus therapy in sepsis: a systematic review of contemporary data. Crit Care Lond Engl.

[13] Schnell D, Camous L, Guyomarc’h S, Duranteau J, Canet E, Gery P (2013). Renal perfusion assessment by renal doppler during fluid challenge in sepsis. Crit Care Med [Internet].

[14] Messmer AS, Zingg C, Müller M, Gerber JL, Schefold JC, Pfortmueller CA (2020). Fluid overload and mortality in adult critical care patients—a systematic review and meta-analysis of observational studies*. Crit Care Med [Internet].

[15] Alobaidi R, Morgan C, Basu RK, Stenson E, Featherstone R, Majumdar SR (2018). Association between fluid balance and outcomes in critically Ill children: a systematic review and meta-analysis. JAMA Pediatr.

[16] Casas-Aparicio GA, León-Rodríguez I, Hernández-Zenteno RJ, Castillejos-López M, Alvarado-de la Barrera C, Ormsby CE (2018). Aggressive fluid accumulation is associated with acute kidney injury and mortality in a cohort of patients with severe pneumonia caused by influenza A H1N1 virus. PLoS ONE.

[17] Wang N, Jiang L, Zhu B, Wen Y, Xi XM, Beijing Acute Kidney Injury Trial (BAKIT) Workgroup (2015). Fluid balance and mortality in critically ill patients with acute kidney injury: a multicenter prospective epidemiological study. Crit Care Lond Engl.

[18] Garzotto F, Ostermann M, Martín-Langerwerf D, Sánchez-Sánchez M, Teng J, Robert R (2016). The dose response multicentre investigation on fluid assessment (DoReMIFA) in critically ill patients. Crit Care Lond Engl.

[19] Acheampong A, Vincent JL (2015). A positive fluid balance is an independent prognostic factor in patients with sepsis. Crit Care Lond Engl.

[20] Teixeira C, Garzotto F, Piccinni P, Brienza N, Iannuzzi M, Gramaticopolo S (2013). Fluid balance and urine volume are independent predictors of mortality in acute kidney injury. Crit Care Lond Engl.

[21] Vaara ST, Korhonen AM, Kaukonen KM, Nisula S, Inkinen O, Hoppu S (2012). Fluid overload is associated with an increased risk for 90-day mortality in critically ill patients with renal replacement therapy: data from the prospective FINNAKI study. Crit Care Lond Engl.

[22] Marik PE, Linde-Zwirble WT, Bittner EA, Sahatjian J, Hansell D (2017). Fluid administration in severe sepsis and septic shock, patterns and outcomes: an analysis of a large national database. Intensive Care Med.

[23] Berthelsen RE, Perner A, Jensen AK, Jensen JU, Bestle MH (2018). Fluid accumulation during acute kidney injury in the intensive care unit. Acta Anaesthesiol Scand.

[24] Woodward CW, Lambert J, Ortiz-Soriano V, Li Y, Ruiz-Conejo M, Bissell BD (2019). Fluid overload associates with major adverse kidney events in critically ill patients with acute kidney injury requiring continuous renal replacement therapy. Crit Care Med.

[25] Hjortrup PB, Haase N, Bundgaard H, Thomsen SL, Winding R, Pettilä V (2016). Restricting volumes of resuscitation fluid in adults with septic shock after initial management: the CLASSIC randomised, parallel-group, multicentre feasibility trial. Intensive Care Med.

[26] Raimundo M, Crichton S, Martin J, Syed Y, Varrier M, Wyncoll D (2015). Increased fluid administration after early acute kidney injury is associated with less renal recovery. Shock Inj Inflamm Sepsis Lab Clin Approaches [Internet].

[27] Inkinen N, Selander T, Pettilä V, Valkonen M, Bäcklund M, Wennervirta J (2020). Noninterventional follow-up vs fluid bolus in RESPONSE to oliguria: the RESPONSE trial protocol and statistical analysis plan. Acta Anaesthesiol Scand.

[28] Singer M, Deutschman CS, Seymour CW, Shankar-Hari M, Annane D, Bauer M (2016). The third international consensus definitions for sepsis and septic shock (sepsis-3). JAMA.

[29] Kellum JA, Lameire N, Aspelin P, Barsoum RS, Burdmann EA, Goldstein SL (2012). Kidney disease: improving global outcomes (KDIGO) acute kidney injury work group. KDIGO clinical practice guideline for acute kidney injury. Kidney Int Suppl [Internet].

[30] Silbert BI, Ho KM, Lipman J, Roberts JA, Corcoran TB, Morgan DJ (2016). Determinants of urinary output response to IV furosemide in acute kidney injury: a pharmacokinetic/pharmacodynamic study. Crit Care Med.

[31] Kahan BC, Morris TP (2012). Reporting and analysis of trials using stratified randomisation in leading medical journals: review and reanalysis. BMJ.

[32] Nunes TSO, Ladeira RT, Bafi AT, de Azevedo LCP, Machado FR, Freitas FGR (2014). Duration of hemodynamic effects of crystalloids in patients with circulatory shock after initial resuscitation. Ann Intensive Care [Internet].

[33] Aya HD, Ster IC, Fletcher N, Grounds RM, Rhodes A, Cecconi M (2016). Pharmacodynamic analysis of a fluid challenge. Crit Care Med [Internet].

[34] Md Ralib A, Pickering JW, Shaw GM, Endre ZH (2013). The urine output definition of acute kidney injury is too liberal. Crit Care [Internet].

[35] Vaara ST, Ostermann M, Bitker L, Schneider A, Poli E, Hoste E (2021). Restrictive fluid management versus usual care in acute kidney injury (REVERSE-AKI): a pilot randomized controlled feasibility trial. Intensive Care Med.

[36] Pickkers P, Darmon M, Hoste E, Joannidis M, Legrand M, Ostermann M (2021). Acute kidney injury in the critically ill: an updated review on pathophysiology and management. Intensive Care Med [Internet].

[37] Sirota JH, Baldwin DS, Villarreal H (1950). Diurnal variations of renal function in man. J Clin Invest [Internet].

