# Supplementary material for: Non-interventional follow-up versus fluid bolus in RESPONSE to oliguria in hemodynamically stable critically ill patients: a randomized controlled pilot trial
Source: Crit Care. 2022 Dec 22;26:401. doi: 10.1186/s13054-022-04283-8 (PMC9773608; doi:10.1186/s13054-022-04283-8)
Supplement: Supplementary file 1 — Additional file 1. Supplemental Tables and Figures. [file 13054_2022_4283_MOESM1_ESM.docx]

Electronic supplementary material

**Non-interventional follow-up versus fluid bolus in RESPONSE to oliguria in hemodynamically stable critically ill patients – a randomized controlled trial**

Nina Inkinen^1,2^, Ville Pettilä^2^, Miia Valkonen^2^, Maija Serlo^2^, Minna Bäcklund^2^, Johanna Hästbacka^2^ Anni Pulkkinen^1^, Tuomas Selander^3^, Suvi T. Vaara^2^

¹ Department of Anesthesia and Intensive Care, Central Finland Hospital Nova, Central Finland Health Care District, Hoitajantie 3, 40620 Jyväskylä, Finland

² Division of Intensive Care Medicine, Department of Perioperative, Intensive Care and Pain Medicine, University of Helsinki and Helsinki University Hospital, Helsinki, Finland

^3^ Science Service Center, Kuopio University Hospital, Kuopio, Finland.

Corresponding author: Nina Inkinen, staff specialist

Address: Department of Anesthesia and Intensive Care, Central Finland Hospital Nova, Hoitajantie 3, 40620 Jyväskylä, Finland

Phone + E-mail: +358 14 269 5375, [nina.inkinen@ksshp.fi](mailto:nina.inkinen@ksshp.fi), ORCID 0000-0002-2551-9322

Index Page

1. Table S1 Detailed inclusion and exclusion criteria 3
2. Table S2 Definitions for protocol violations and adverse events 5
3. Table S3 Primary and secondary outcomes in the crude population 6
4. Table S4 Patient characteristics in fluid bolus group patients 7
5. Figure S1 Exploratory outcomes – Central minus peripheral temperature, pH and base excess 10
6. Table S5 Non-physiological exploratory outcomes 11
7. Table S6 Primary and secondary outcomes among septic patients 12
8. Table S7 Primary and secondary outcomes among patients with acute kidney injury 13

9. Table S8 Primary and secondary outcomes in the per protocol population 14

1. Table S1 Detailed inclusion and exclusion criteria

| **Inclusion criteria (all must be fulfilled)** | |
| --- | --- |
| Age over 18 | o on the randomization day |
| Emergency admission to an ICU | o elective admissions (for example after elective surgery) are not eligible |
| Mean arterial pressure (MAP) >65 mmHg (with vasopressors if needed) and initial fluid resuscitation  (over 20mL/kg iv-fluids during the last 12hrs) for shock/hypovolemia has been given OR patient has  been in the ICU over 6hrs | o Iv-fluids includes crystalloids and blood products, but not carrier fluids for medication |
| Oliguria (urine output less than 0.5mL/kg/h) for at least 2 consecutive hours | o Patient body weight is that registered on ICU admission |
| **Exclusion criteria (none cannot be present)** | |
| Marked fluctuations in hemodynamics within the last 2 hours pre-randomization (cardiac  arrhythmias affecting blood pressure, increase in norepinephrine need over 0.2 microg/kg/min, need for initiation of inotrope/inodilator) |  |
| Administration of furosemide within last 6 hours |  |
| Chronic kidney disease (estimated pre-critical illness GFR < 60mL/min/1.73m2) |  |
| Renal replacement therapy (RRT) | o RRT has been already started in the ICU for AKI  o Commencing RRT (according to last laboratory values) is likely within the next 6hrs  o Patient undergoes regular (chronic) dialyses  o Patient has a history of kidney transplantation |
| Urgent indications for commencing RRT for AKI are present (based on last blood work) | o plasma potassium > 6mmol/L  o severe metabolic acidosis (pH<7.20 and bicarbonate <12mmol/L),  o evidence of severe respiratory failure (PaO2/FiO2 ratio <200) and clinical perception of  volume overload  o AKI has continued over 72hrs (creatinine remains more than twice the normal level/oliguria continues) |
| Fluid overload (cumulative fluid accumulation exceeds 10% of baseline body weight) | o Even if fluid overload has no impact on oxygenation |
| Pulmonary edema (bilateral infiltrates in chest x-ray) |  |
| Active bleeding (need for transfusion, platelets, or fresh frozen plasma) | o operational definition: transfusion is planned within next 6 hrs |
| Suspected or known intra-abdominal hypertension (intra-abdominal pressure >16mmHg) |  |
| Pregnant or lactating | o clinical team being aware of pregnancy/lactation |
| Expected survival less than 24h | o Patients whose ICU treatment is withdrawn  o Strong suspicion that patient will not survive over 24hrs  o Organ donors |
| Obtaining informed written consent is not possible / consent is denied |  |

2. Table S2 Definitions for protocol violations and adverse events

| **Protocol violations** | Patient received furosemide or other diuretic during the 2-hour period |
| --- | --- |
|  | Changing mean arterial pressure (MAP) target (defined as MAP level increased from baseline target AND increased vasopressor dose from baseline) |
|  | No full 500mL bolus in fluid bolus group |
|  | Fluid bolus (other than rescue bolus) in the follow-up group |
|  | Erroneously randomized non-eligible patient* |
| **Adverse events** | Assessed from randomization until next morning |
|  | Defined as events reported by the treating clinician in the open questionnaire included in the protocol |

* Patient recruitment was continued beyond the 130 randomized patients to have erroneously randomized non-eligible patients replaced. We randomized patients mainly during last 10 minutes before the beginning of the 2-hour study period. Unexpectedly, the urine output exceeded 0.5mL/kg/h in the end of the last hour after randomization but before the beginning of 2-hour study period in five patients, and these patients were considered erroneously randomized. In two patients, the urinary catheter was noticed to be incorrect place and the amount diuresis could not be confirmed reliably. These patients were considered erroneously randomized.

3. Table S3. Primary and secondary outcomes in the crude population

|  | Data available | Follow-up group | Fluid bolus group | Follow-up group vs Fluid bolus group (95% CI) | P-value |
| --- | --- | --- | --- | --- | --- |
| Doubled urine output (%) | 130 | 10 (15.9) | 22 (32.9) | 0.48* (0.22 – 0.45) | 0.032 |
| Change in individual urine output, median [IQR], mL | 130 | -7 [-19 – 17] | 19 [0 – 53] | -26** (-41 - -10) | <0.001 |
| Duration of consecutive oliguria,^a^ median [IQR], hrs | 130 | 4 [2 – 8] | 2 [0 – 6] | 2** (0-4) | 0.060 |
| Cumulative fluid balance 6hrs post-randomization, median [IQR], mL^b^ | 128 | 678 [518 – 1029] | 1071 [822 – 1505] | -393** (-591 - -238) | <0.001 |

^a^ Urine output <0.5mL/kg/h, data collected to 30d post-randomization or ICU discharge if earlier

^b^ Including fluid input and urine output

*Risk ratio

**Median difference

1. Table S4 Patient characteristics in fluid bolus group patients

|  | Patients, who did not double urine output, n=45 | Patients, who doubled urine output, n=22 |
| --- | --- | --- |
| Age (years) | 69 [59-75] | 70 [60-76] |
| Sex; female (%) | 17 (37.8) | 9 (40.9) |
| Weight (kg) | 82 [75-100] | 86 [71-100] |
| Hypertension (%) | 31 (68.9) | 13 (59.1) |
| Chronic heart failure (%) | 2 (4.4) | 0 (0.0) |
| Atrial fibrillation (%) | 11 (24.4) | 3 (13.6) |
| Coronary artery disease (%) | 5 (11.1) | 2 (9.1) |
| Arteriosclerosis obliterans (%) | 4 (8.9) | 1 (4.5) |
| Chronic obstructive pulmonal disease (%) | 3 (6.7) | 1 (4.5) |
| Chronic liver insufficiency (%) | 0 (0.0) | 0 (0.0) |
| Diabetes (%) | 14 (31.1) | 8 (36.4) |
| Malignancy (%) | 8 (17.8) | 1 (4.5) |
| Rheumatoid disease (%) | 3 (6.7) | 1 (4.5) |
| SAPS II score | 42 [33-52] | 38 [33-46] |
| Surgical admission (%) | 27 (60.0) | 10 (45.5) |
| **At randomization** |  |  |
| Time from ICU admission to randomization (hours) | 20.9 [13.9-34.8] | 18.3 [11.0-39.6] |
| Sepsis (%) | 27 (60.0) | 10 (45.5) |
| Acute kidney injury (%)* | 30 (66.7) | 10 (45.5) |
| Invasive ventilation (%) | 21 (46.7) | 13 (59.1) |
| Vasoactive medication (%)** | 22 (48.9) | 10 (45.5) |
| Continuous sedation (%) | 18 (40.0) | 10 (45.5) |
| Cumulative balance from ICU admission to randomization (mL) | 2263 [1570-3803] | 1569 [954-3346] |
| SOFA score at randomization (-24hrs to 0h) | 7 [6-9] | 7 [4-10] |
| SOFA score 24hrs post randomization (0h to 24hrs) | 6 [4-10] | 7 [2-8] |

ICU, intensive care unit

SAPS, Simplified acute physiology score II

SOFA, sequential organ failure assessment; considering all six organ systems

* According to KDIGO criteria

**Norepinephrine, dobutamine, epinephrine, levosimendan, milrinone, vasopressin, dopamine or other

***Data missing from one patient

Data included from 67 patients in fluid bolus group

Categorical data reported as count (percentage) and continuous data as median [interquartile range, IQR]

5. Figure S1 Exploratory outcomes – Central minus peripheral temperature (a), pH (b), and base excess (c)


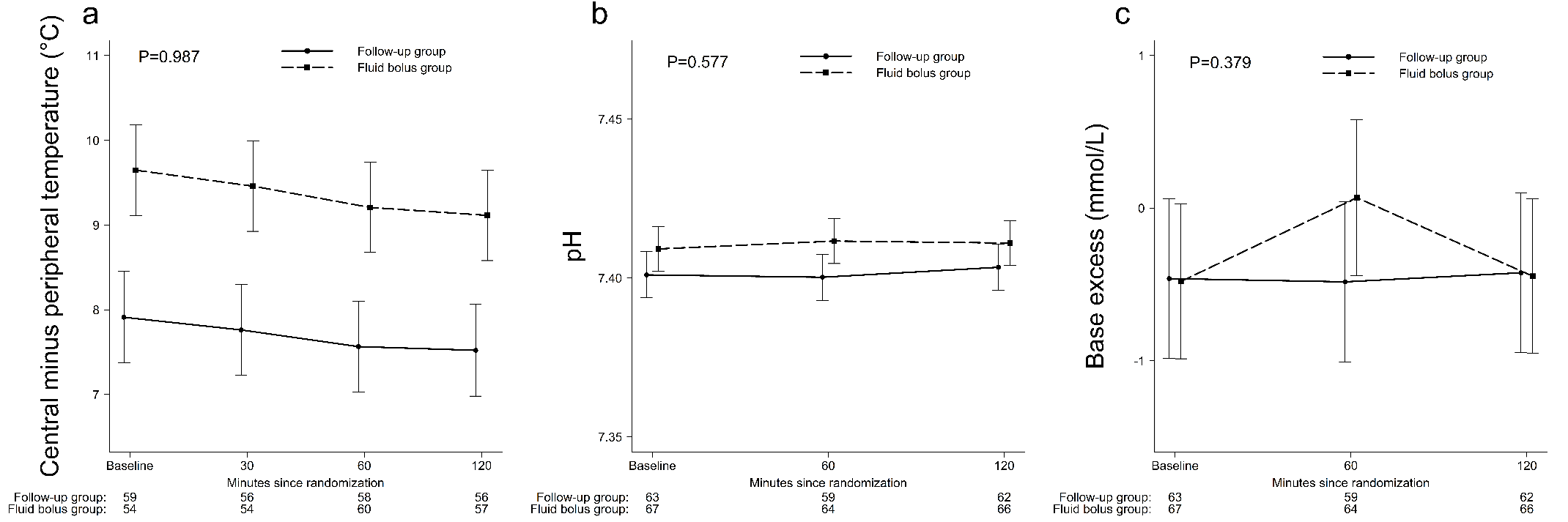


The difference between follow up and fluid bolus group was compared with median regression.

6. Table S5 Non-physiological exploratory outcomes

|  | Data available | Follow-up group, n=63 | Fluid bolus group, n=67 | Follow-up group vs Fluid bolus group (95% CI)^d^ | P-value |
| --- | --- | --- | --- | --- | --- |
| Number of patients receiving a rescue bolusᵃ (%) | 130 | 1 (1.6) | 0 (0.0) | 3.19 (0.13 – 76.83) | 0.485 |
| Number of patients with protocol violationsᵇ | 130 | 2 (3.2) | 5 (7.5) | 0.44 (0.06 – 1.91) | 0.302 |
| Number of patients with adverse eventsᶜ | 130 | 1 (1.6) | 0 (0.0) | 3.19 (0.13 – 76.83) | 0.485 |
| Highest AKI stage within 24h, median [IQR), n=91 | 128 | 1 [0-1] | 1 [0-2] | 0 (0-0) | 0.861 |
| Highest AKI stage within 48h, median [IQR], n=90 | 126 | 1 [0-2] | 1 [0-2] | 0 (0-0) | 0.800 |
| Highest AKI stage during ICU stay, median [IQR], n=95 | 128 | 1 [0-2] | 1 [0-2] | 0 (0-0) | 0.932 |
| Number of patients receiving RRT | 130 | 9 (14.3) | 7 (10.4) | 1.38 (0.57 – 3.57) | 0.477 |

ᵃ Within 2-hour study period

ᵇ Protocol violations included 1 patient because of receiving a fluid bolus (other than rescue bolus) in the follow-up group, 1 non-eligible patient because of erroneously randomized, 2 patients because of receiving fluid bolus in fluid bolus group that was larger than 500 mL, and 3 patients because of receiving unplanned other fluid during the 2-hour study period

ᶜ One patient suffered from unplanned extubation

^d^ Adjusted with sepsis and acute kidney injury except number of patients receiving rescue bolus and number of patients with protocol violations due to low number of observations

AKI; Acute kidney injury, ICU; Intensive care unit, IQR; interquartile range, RRT; Renal replacement therapy

7. Table S6 Primary and secondary outcomes among septic patients

|  | Data available | Follow-up group, n=35 | Fluid bolus group, n=37 | Follow-up group vs Fluid bolus group (95% CI) | P-valueᶜ |
| --- | --- | --- | --- | --- | --- |
| Doubled urine output (%) | 72 | 5 (14.3) | 10 (27.0) | 0.54* (0.19 – 1.34) | 0.198 |
| Change in individual urine output, median [IQR], mL | 72 | 0 [-31 – 17] | 10 [-6 – 40] | -18** (-31 - 3) | 0.079 |
| Duration of consecutive oliguriaᵃ, median [IQR], hrs | 72 | 4 [2 – 11] | 4 [1 -10] | -1** (-4 – 4) | 0.757 |
| Cumulative fluid balance 6hrs post-randomization, median [IQR], mLᵇ | 71 | 672 [591 – 915] | 1222 [995 – 1663] | -504** (-830 - -310) | <0.001 |

ᵃ Urine output <0.5mL/kg/h, data collected to 30d post-randomization or ICU discharge if earlier

ᵇ Including fluid input and diuresis

ᶜ Adjusted with AKI

*Risk ratio

**Median difference

8. Table S7 Primary and secondary outcomes among patients with acute kidney injury

|  | Data available | Follow-up group, n=37 | Fluid bolus group, n=40 | Follow-up group vs Fluid bolus group (95% CI) | P-valueᶜ |
| --- | --- | --- | --- | --- | --- |
| Doubled urine output (%) | 77 | 5 (13.5) | 10 (25.0) | 0.56* (0.19 – 1.40) | 0.222 |
| Change in individual urine output, median [IQR], mL | 77 | -9 [-28 – 13] | 10 [-7 – 40] | -20** (-33 - 0) | 0.042 |
| Duration of consecutive oliguriaᵃ, median [IQR], hrs | 77 | 4 [2 – 14] | 4 [1 – 13] | 0** (-5 - 4) | 0.759 |
| Cumulative fluid balance 6hrs post-randomization, median [IQR], mLᵇ | 76 | 692 [520 – 949] | 1090 [800 – 1570] | -351** (-720 - -143) | 0.001 |

ᵃ Urine output <0.5mL/kg/h, data collected to 30d post-randomization or ICU discharge if earlier

ᵇ Including fluid input and diuresis

ᶜ Adjusted with sepsis

*Risk ratio

**Median difference

1. Table S8. Primary and secondary outcomes in the per protocol population

|  | Data available | Follow-up group | Fluid bolus group | Follow-up group vs Fluid bolus group (95% CI) | P-value |
| --- | --- | --- | --- | --- | --- |
| Doubled urine output (%) | 122 | 8 (13.3) | 19 (30.6) | 0.43* (0.19 – 0.86) | 0.025 |
| Change in individual urine output, median [IQR], mL | 122 | -8 [-18 – 15] | 18 [0 – 50] | -22** (-35 - -10) | <0.001 |
| Duration of consecutive oliguriaᵃ, median [IQR], hrs | 122 | 4 [2-8] | 2 [0-6] | 2** (0-4) | 0.066 |
| Cumulative fluid balance 6hrs post-randomization, median [IQR], mLᵇ | 120 | 656 [511 – 887] | 1059 [819 – 1391] | -376** (-629 - -237) | <0.001 |

ᵃ Urine output <0.5mL/kg/h, data collected to 30d post-randomization or ICU discharge if earlier

ᵇ Including fluid input and diuresis

*Risk ratio

**Median difference
